# Frequency Shifts and Noncoincidence Effect of the CN Stretching Modes and the Solvation Structures of Acetonitrile Electrolyte Solutions

**DOI:** 10.1021/acs.jpcb.5c01894

**Published:** 2025-07-07

**Authors:** Miyu Hirose, Yukichi Kitamura, Hideaki Shirota, Hajime Torii

**Affiliations:** 1 Applied Chemistry and Biochemical Engineering Course, Department of Engineering, Graduate School of Integrated Science and Technology, 13058 Shizuoka University, 3-5-1 Johoku, Chuo-ku, Hamamatsu 432-8561, Japan; 2 Department of Chemistry, 12737 Chiba University, 1-33 Yayoi, Inage-ku, Chiba 263-8522, Japan; 3 Department of Optoelectronics and Nanostructure Science, Graduate School of Science and Technology, 13058 Shizuoka University, 3-5-1 Johoku, Chuo-ku, Hamamatsu 432-8561, Japan

## Abstract

Electrolyte solutions often show characteristic spectral features in their vibrational spectra, and a quantitative analysis of them is a key toward better understanding of the solvation structures and intermolecular interactions based on those spectral features. Here, such an analysis is carried out for the C≡N stretching mode of some electrolyte solutions of acetonitrile. It is found that a negative noncoincidence effect, in which the isotropic Raman component is located at a higher frequency than the anisotropic component, is observed for the band that newly appears upon solvation of salt, and based on a theoretical analysis that refers to quantum chemical calculations and molecular dynamics simulation results, it is shown that this phenomenon arises from the vibrational coupling among the C≡N bonds clustering around each metal ion (Li^+^, Mg^2+^, Ca^2+^, or Zn^2+^). The transition dipole coupling mechanism reasonably well explains the sign and magnitude of this intermolecular vibrational coupling. The overall high-frequency shift of this newly appearing band (compared to the band of neat liquid) is largely explained by an electrostatic interaction model that takes into account the spatially nonuniform nature of the electrostatic potentials and fields operating from each metal ion on the solvent molecules around it. On the basis of these results, the relation between the spectral features and the solvation structures is discussed.

## Graphical abstract

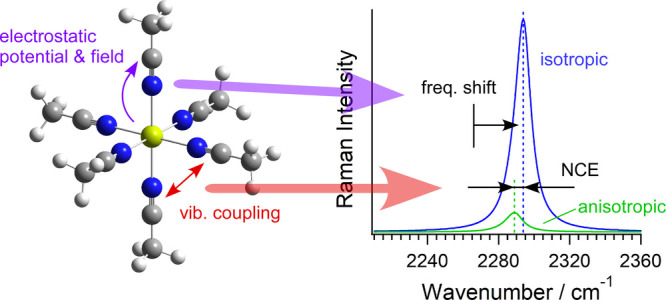

## 1 Introduction

Acetonitrile is known as a dipolar solvent with a large molecular dipole moment and a rather large relative permittivity,^1,2^ and as such, it dissolves many organic and inorganic salts. In practical terms, acetonitrile electrolyte solutions have attracted great attention due to their promising applicability as media in rechargeable batteries.^3–9^ For deeper understanding of their structural characteristics, the solvation structures around metal ions have been examined by neutron diffraction and X-ray spectroscopies.^10–12^ Vibrational spectroscopy is also useful in this context^13^ because acetonitrile has the C≡N stretching mode, which is a marker mode that is sensitive to intermolecular interactions and structural configurations.^14^ It is known that this mode shows low-frequency shifts upon dipolar solvation^15–20^ but high-frequency shifts upon hydrogen-bond formation.^16–23^ It has been elucidated that these frequency shifts of opposite signs are both explainable by an electrostatic interaction model that contains the scalar and vector components and, thus, takes into account the spatially inhomogeneous (or nonuniform) nature of the electrostatic situation generated by hydrogen-bond donating molecules.^24–26^

High-frequency shifts of the C≡N stretching mode of acetonitrile are also observed upon interactions with metal ions.^8,9,27–39^ Considering that the electric charge of each metal ion also gives rise to a nonuniform electric field on the C≡N bonds clustering around it, it would be reasonable to expect that those frequency shifts can reasonably be explained by the same or similar electrostatic interaction model, but a quantitative analysis is needed to deepen our understanding of the extent to which the model can actually reproduce the observed frequency shifts. In a previous study,^39^ it was suggested that the frequency shifts depend on the (surface) charge densities of the metal ions. This seems to be consistent with the above-mentioned idea that the electrostatic interactions play a major role in the frequency shifts, but because typical ionic radii (e.g., 0.86 Å for Mg^2+^)^40^ are rather far from the ion–ligand distances [e.g., ∼2.2 Å for Mg^2+^···N­(≡C)],^10^ it would be preferable to quantitatively examine the relations among the frequency shifts, the structural configurations such as the coordination number of the solvent molecules clustering around each metal ion, and the electric fields and/or electrostatic potentials operating on those solvent molecules on a well-founded theoretical basis.

It is known that some vibrational modes are coupled strongly between molecules in condensed phases, and a manifestation of those intermolecular vibrational couplings is the noncoincidence effect (NCE),^41–45^ which is a spectroscopic phenomenon that the isotropic and anisotropic components of a band in a polarized Raman spectrum appear at different frequency positions (defined as δν_NCE_ ≡ ν_aniso_ – ν_iso_). Sometimes it also refers to the frequency difference between the infrared (IR) and isotropic Raman components (ν_IR_ – ν_iso_). An important point in the context of the present study is that, for the electrolyte solutions of acetone and carbonate esters, sign inversion of the NCE has been observed in the C=O stretching band; the NCE is positive in neat liquid and is negative for a band that appears newly upon solvation of salt.^46–48^ It has been discussed that this sign-inverted NCE arises from the vibrational coupling between the molecules clustering around a metal ion. The structural configurations of those molecules (atoms with partial negative charges clustering around a metal ion) are totally different from those in neat liquid (typically parallel head-to-tail or antiparallel side-by-side because of the dipolar nature of the liquid), and based on them, sign inversion of the intermolecular vibrational coupling is predicted by the transition dipole coupling (TDC) mechanism. As a result, information on the solvation structures is developed along the frequency axis rather than along the intensity axis of the spectra.^48^ In the case of the C≡N stretching mode of acetonitrile, the observed NCE is rather small (∼1 cm^–1^) in neat liquid,^49^ but it would be interesting to see whether a similar phenomenon is observed for electrolyte solutions of acetonitrile and, if it is really observed, whether it is possible to interpret it consistently to support the discussion on the solvation structures based on the above-mentioned overall high-frequency shifts.

In this study, we deal with this problem by conducting spectroscopic measurements and theoretical calculations. Polarized Raman spectra are observed in the frequency region of the C≡N stretching mode for the acetonitrile solutions of the bis­(trifluoromethylsulfonyl)­amide (NTf_2_^–^) salts of Li^+^, Mg^2+^, Ca^2+^, and Zn^2+^. Two types of theoretical calculations are carried out: quantum chemical calculations on acetonitrile–metal ion clusters and molecular dynamics (MD) simulations on ideally dilute solutions. Based on those experimental and theoretical results, the extent of validity of the electrostatic interaction model for the frequency shifts and the TDC mechanism for the vibrational coupling as well as the relations between the spectral features and the structural configurations in the solutions will also be discussed.

## 2 Computational and Experimental Procedures

### 2.1 Calculations and Analyses on Acetonitrile–Metal Ion Clusters

Calculations and analyses were carried out for the M^*m*+^(CH_3_CN)_*n*_ clusters (M^*m*+^ = Li^+^, Mg^2+^, Ca^2+^, or Zn^2+^ and *n* = 1–6), and additionally for an isolated CH_3_CN molecule as a reference. The initial structure of each cluster was prepared by making the N atom(s) of the CH_3_CN molecule(s) directly interact with the metal ion at symmetric (for *n* ≥ 2) positions around it (linear for *n* = 2, triangular for *n* = 3, tetrahedral for *n* = 4, trigonal bipyramid for *n* = 5, and octahedral for *n* = 6), with the C≡N bond(s) being set in the radial direction(s), and then, the structure was fully optimized, followed by calculations of the vibrational force constants (on the Cartesian-coordinate basis) and the vibrational frequencies. The B3LYP functional of density functional theory (DFT) and the HF, MP2, and MP3 levels of the ab initio molecular orbital (MO) method were employed in combination with the 6-31+G­(2df,p) basis set. At the MP3 level, because of the limit of the available computational resources, the calculations were limited to the range of *n* = 1–5 (for M^*m*+^ = Li^+^, Mg^2+^, and Ca^2+^) or *n* = 1–4 (for M^*m*+^ = Zn^2+^). In addition, for the same reason, the vibrational frequencies of the C≡N stretching modes at the MP3 level were calculated with the “two coordinates per molecule” (2CpM) approximation, where only the vibrations along the C≡N and C–C stretching internal coordinates were included. In this case, the forces were calculated for displaced structures (by ±0.004 Å) along these coordinates to derive the force constants (on the internal-coordinate basis) by numerical differentiation, and then, the vibrational frequencies were calculated by the traditional GF matrix^50^ method. Since the displacements were made along internal coordinates, it was possible to generate the displaced structures only from the structural properties and atomic masses. The validity of this approximation was checked by the calculations at the B3LYP, HF, and MP2 levels, as described below in Section 3.1.

To estimate an effective electric charge of the M^*m*+^ ion in each cluster that is used in combination with the electrostatic interaction model of frequency shifts, the electron density change occurring upon cluster formation, defined as δρ^(el)^(***r***) ≡ [ρ^(el)^(***r***)]_cluster_ – Σ [ρ^(el)^(***r***)]_molecule_ – [ρ^(el)^(***r***)]_ion_, was calculated for some of the clusters and was compared with the electron density change induced on a CH_3_CN molecule by an electric charge of 0.2 *e* placed at the location of the ion. In addition, to support the discussion on the spatial characteristics of the dipole derivative ∂μ/∂*R*_CN_ of the C≡N stretch (internal coordinate, denoted as *R*_CN_), the electron density derivative ∂ρ^(el)^(***r***)/∂*R*_CN_ was calculated, and its properties were examined. The evaluation points ***r*** of ρ^(el)^(***r***) were taken in a rectangular box with the length of each edge of 17.7–21.0 Å (the exact dimension depending on the structure of each cluster), so that each boundary of the box is at least 5 Å from any atom in the cluster, with an interval of 0.02 Å. The derivative was evaluated by numerical differentiation from ρ^(el)^(***r***) calculated for the equilibrium (*R*_CN_ = 0) and displaced (*R*_CN_ = 0.03 Å) structures.

All the DFT and ab initio MO calculations described above were performed by using the Gaussian 09 program,^51^ and the analyses (including the preprocessing of the DFT and ab initio MO calculations) were done with our original programs.

### 2.2 Molecular Dynamics Simulations of Solutions

Classical MD simulations were carried out, to mimic the systems in the essentially infinite dilution limit, for solutions consisting of a metal ion M^*m*+^ (= Li^+^, Mg^2+^, Ca^2+^, or Zn^2+^), *m* (= 1 or 2) chloride or NTf_2_^–^ counteranion(s), and 140 or more (up to 479) solvent acetonitrile molecules in a rectangular cell. The restrained electrostatic potential (RESP) atomic partial charges of acetonitrile and the NTf_2_^–^ ion were estimated by calculations at the B3LYP/6-31+G­(d,p) or B3LYP/6-31+G­(2df,p) level employing the Merz–Kollman (MK) scheme.^52,53^ The general AMBER force field (GAFF) parameters were used for the nonelectrostatic terms. The time step was set to 1 fs, with the constraint on the C–H bond lengths employing the SHAKE algorithm.^54^ After energy minimization, each system was equilibrated for 2 ns at 300 K and 1 bar (NPT ensemble), after which a production run of 20 ns was carried out.

Semiempirical quantum mechanics/molecular mechanics (QM/MM) MD simulations were also performed for the same set of systems, with the number of solvent acetonitrile molecules being increased to 1400 or more (up to 5586). Starting from the solution structures obtained by classical MD, semiempirical QM/MM MD simulations were carried out at 300 K (NVT ensemble) for 100 ps with a time step of 0.5 fs by using some semiempirical QM models (among PM6, PM3, AM1, MNDO, and DFTB3) where available for each metal ion. The adaptive solvation method^55,56^ was employed to designate the active QM region that contains the metal ion and the nearest 3–4 (for Li^+^) or 6–8 (for the divalent ions) acetonitrile molecules around it and the QM-to-MM transition region that consists of the next nearest 3 acetonitrile molecules.

All the MD simulations described above were performed by using the AMBER 22 program,^57^ and the solution structures were extracted from those results by using the CPPTRAJ program.^58^ The subsequent spectral simulations (described in Section 3.3) were done with our original programs.

### 2.3 Experimental Procedure

Lithium bis­(trifluoromethylsulfonyl)­amide (LiNTf_2_; 99.7%, Kanto), magnesium­(II) bis­(trifluoromethylsulfonyl)­amide (Mg­(NTf_2_)_2_; >97%, TCI), calcium­(II) bis­(trifluoromethylsulfonyl)­amide (Ca­(NTf_2_)_2_; >98%, TCI), zinc­(II) bis­(trifluoromethylsulfonyl)­amide (Zn­(NTf_2_)_2_; >97%, TCI), and acetonitirle-*d*_3_ (CD_3_CN; >99%, 99.8%D, TCI) were used as received. The reason why CD_3_CN, instead of normal acetonitrile CH_3_CN, was used in this study is because a spectral splitting occurs in CH_3_CN due to Fermi resonance, which leads to a complexity in assigning the observed Raman bands.^30,33,39^ The salt concentration of the solution was kept at 1.00 mol kg^–1^.

A laboratory-built steady-state Raman spectrometer setup was employed to measure Raman spectra, and the details of the apparatus have been reported elsewhere.^59^ In this study, a diode laser (RGB Lasersystems, Lambda Beam 633–70WL) with an output power of 70 mW at 632.85 nm (≤0.4 nm full width at half-maximum) and the horizontal polarization was used. The polarization of the light was rotated to the vertical using a λ/2 plate before irradiating the sample. The scattered light passed through a polarizer, then a depolarizer, and a sharp cut long-pass filter. The passed scattered light was then focused on the slit of a spectrograph (Andor, SR-500i-B1-R with a grating SR5-GRT-1200-0600) via an achromatic lens. The polarizer was set at vertical (V) for the VV-polarized spectrum and horizontal (H) for the VH-polarized (also called depolarized or anisotropic component) spectrum. A charge-coupled device camera (Andor, DU420ABEX2-DD) was used as a detector. To record a spectrum, the accumulation of 3000 times with an exposure time of 200 ms was performed. The wavelength for the Raman spectrometer was calibrated using a neon lamp, and the intensity was corrected using a halogen lamp. The resolution of the Raman spectrometer setup was approximately 1.8 cm^–1^. Before the Raman spectral measurements, the sample solutions were injected into a 1 cm optical-path length quartz cell using a 0.02 μm pore size Anotop filter (Whatman). A Peltier temperature controller (Quantum Northwest, Luma 40) was used to keep the sample temperature at 298.0 ± 0.1 K.

## 3 Results and Discussion

### 3.1 Validity of the “Two Coordinates per Molecule” (2CpM) Approximation

At the MP3/6-31+G­(2df,p) level, because of the limit of available computational resources, the calculations of the vibrational frequencies of the C≡N stretching modes were required to be carried out efficiently but (of course) with sufficient accuracy. In the present study, we adopted the 2CpM approximation, where only the vibrations along the C≡N and C–C stretching internal coordinates were included in the calculations. The validity of this approximation was checked by doing calculations with this approximation at the B3LYP, HF, and MP2 levels and by comparing the calculated frequencies with those derived from the full treatment of the vibrational force constants. In this case, the vibrational force constant matrix on the Cartesian-coordinate basis obtained directly from the DFT or ab initio MO calculation was transformed to that on the internal-coordinate basis, the elements related to the C≡N and C–C stretching internal coordinates were extracted to form a force constant matrix of size 2*n* × 2*n*, and then it was combined with the G matrix (also of size 2*n* × 2*n*) in the traditional GF matrix method to derive the vibrational frequencies of the C≡N stretching modes. The results with regard to the IR, isotropic Raman, and anisotropic Raman intensity-weighted average frequencies (ν_IR_, ν_iso_, and ν_aniso_) are shown in Figure 1a–c. It is clearly seen that in all cases the 2CpM approximation works well. There are small systematic deviations with average sizes of 2.59, 1.65, and 3.09 cm^–1^ at the B3LYP, HF, and MP2 levels, respectively, but they are so systematic that the shifts from the frequency of an isolated CH_3_CN molecule are affected to a significantly less extent (−0.54, −0.67, and −1.46 cm^–1^, respectively). In calculating the values of NCE (ν_IR_ – ν_iso_ and ν_aniso_ – ν_iso_), the systematic deviations are also almost totally canceled as shown in Figure 1d–f. As a result, it is concluded that the 2CpM approximation can be safely used in calculating the C≡N stretching frequencies of the M^*m*+^(CH_3_CN)_*n*_ clusters.

**Figure 1:**
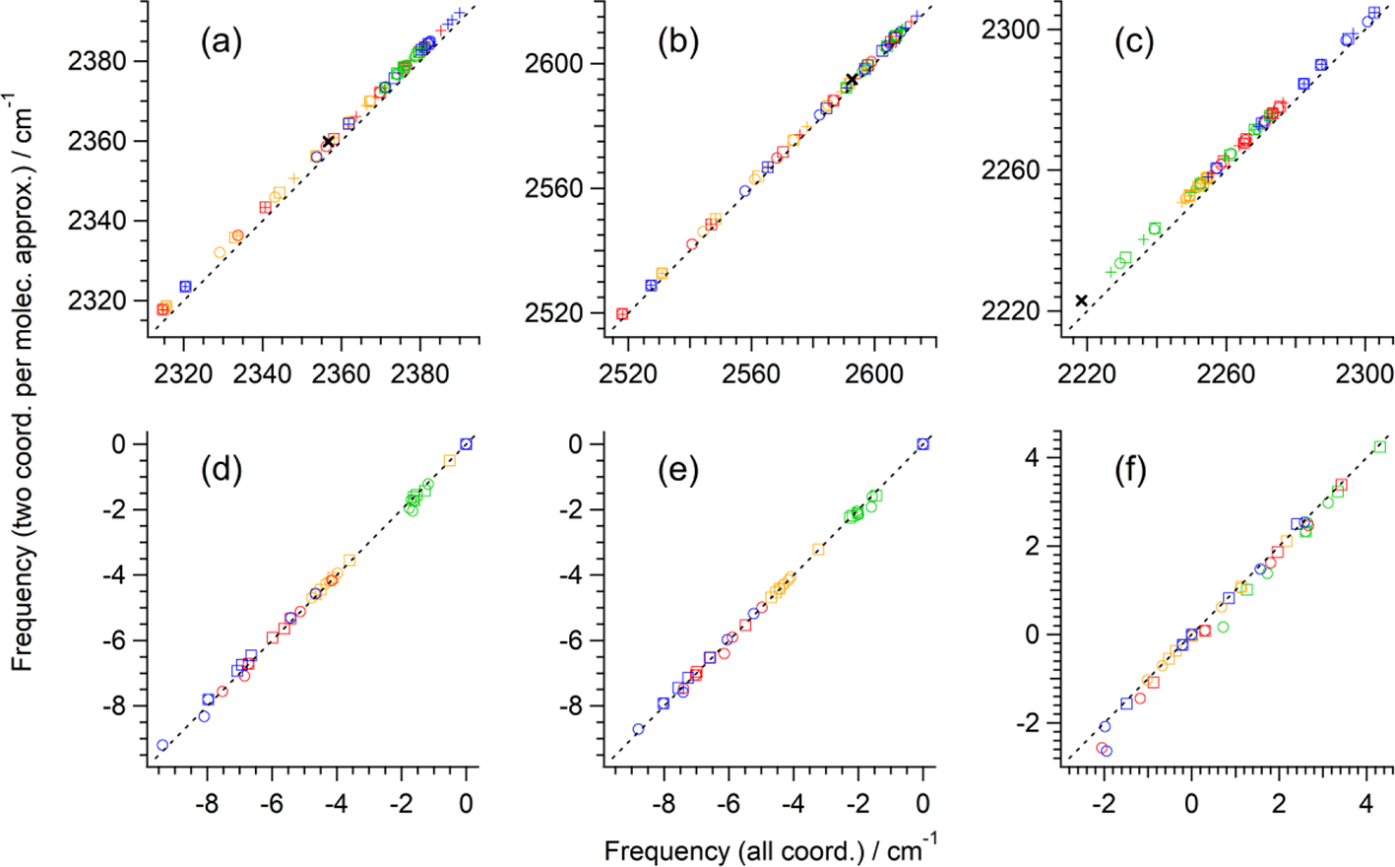
(a–c) Intensity-weighted average frequencies of the C≡N stretching normal modes of the M^*m*+^(CH_3_CN)_*n*_ clusters (M^*m*+^ = Li^+^, Mg^2+^, Ca^2+^, or Zn^2+^ and *n* = 1–6) and an isolated CH_3_CN molecule calculated by including only the C≡N and C–C stretching internal coordinates (2CpM approximation) plotted against those calculated by including all coordinates. (d–f) Corresponding values of the NCE calculated as ν_IR_ – ν_iso_ or ν_aniso_ – ν_iso_. Circle: IR (or ν_IR_ – ν_iso_), square: anisotropic Raman (or ν_aniso_ – ν_iso_), + : isotropic Raman, green: Li^+^, red: Mg^2+^, orange Ca^2+^, blue: Zn^2+^, × (black): isolated molecule. Calculated at the (a,d) B3LYP/6-31+G­(2df,p), (b,e) HF/6-31+G­(2df,p), and (c,f) MP2/6-31+G­(2df,p) levels. The black dotted line in each panel indicates the line of gradient unity passing through the origin drawn as a guide to the eye.

Because the C≡N stretching mode is rather isolated in frequency from the other modes, one might think that it is sufficient to include the G and F matrix elements of only the C≡N stretching internal coordinate for estimating the C≡N stretching frequency [“one coordinate per molecule” (1CpM) approximation]. In fact, this is not the case, as shown in Figure S1 in the Supporting Information. For example, for an isolated CH_3_CN molecule, the C≡N stretching frequency estimated within the 1CpM approximation is deviated by −98.3, −96.2, and −124.5 cm^–1^ at the B3LYP, HF, and MP2 levels, respectively, from that obtained by full treatment. In addition, the deviations calculated for the clusters are rather scattered. This result means that the intramolecular coupling with the vibration(s) along the C–C stretching internal coordinate(s) should be taken into account for sufficiently correct estimation of the C≡N stretching frequencies. As a result, we have adopted the 2CpM approximation rather than the 1CpM approximation in the calculations at the MP3 level.

### 3.2 Frequency Shifts and Solvation Structures around Metal Ions

The shifts of the IR and isotropic Raman intensity-weighted average frequencies (δν_IR_ and δν_iso_) of M^*m*+^(CH_3_CN)_*n*_ from that of an isolated CH_3_CN molecule calculated at the B3LYP, HF, MP2, and MP3 levels are plotted against *n* in Figure 2a–h. In the case of δν_IR_ at B3LYP shown in panel a, except for the Li^+^ clusters, the frequency increases as *n* increases, and positive frequency shifts are predicted for large *n*. Positive frequency shifts are also predicted for the Li^+^ clusters irrespective of the value of *n*. In comparing these frequency shifts with those observed experimentally, one should note that the C≡N bonds in the calculated M^*m*+^(CH_3_CN)_*n*_ clusters are strongly interacting with M^*m*+^ as in the real solvation structures in solution, but the C≡N bond of an isolated CH_3_CN molecule is totally free from intermolecular interactions in contrast to the situation in the liquid phase. Therefore, we have taken the observed gas-to-liquid frequency shift (−17.5 cm^–1^)^60^ as a reference and have plotted it as a horizontal dotted line in panels a–h.

**Figure 2:**
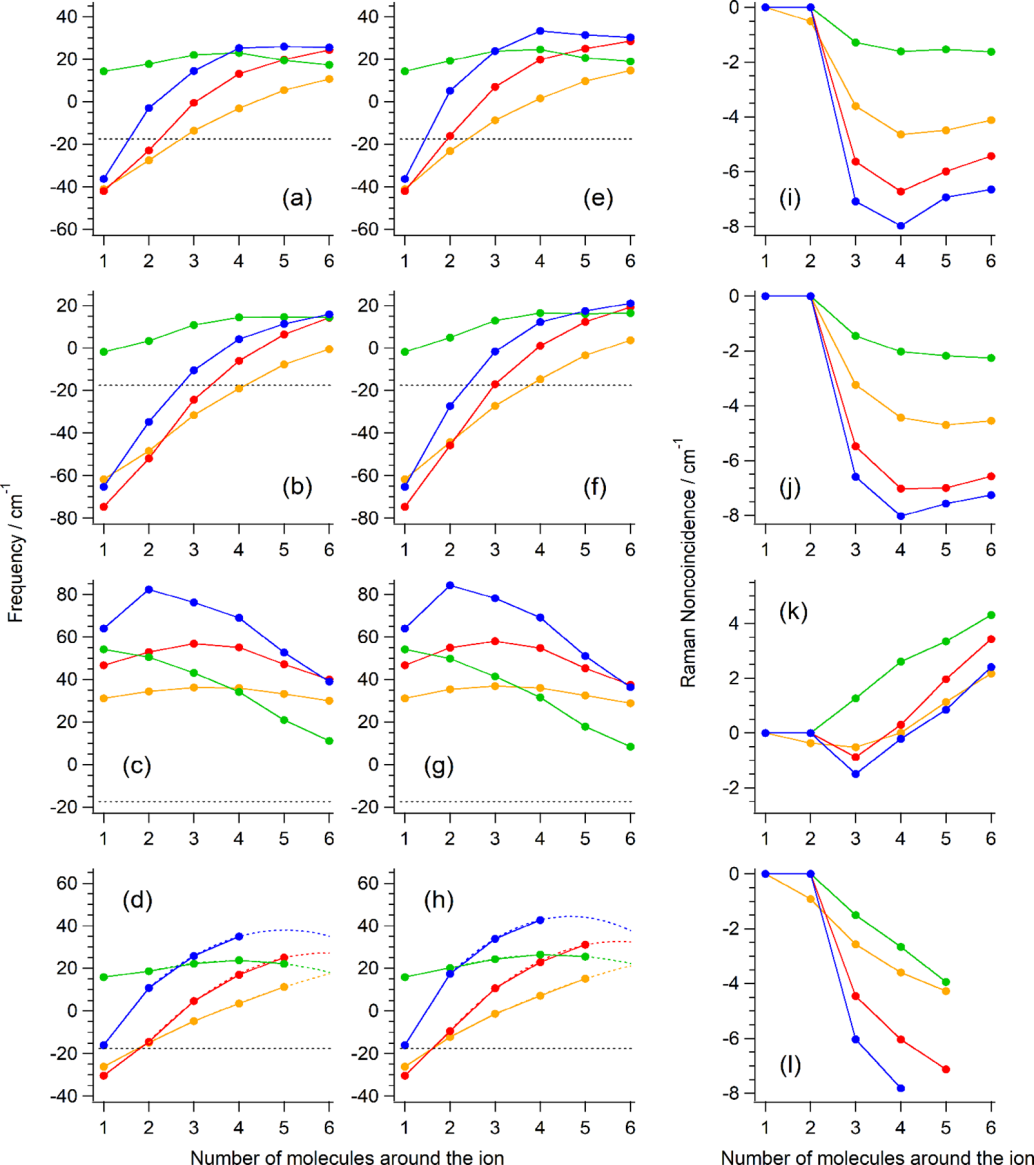
(a–d) Shifts of the IR intensity-weighted average frequencies of the C≡N stretching normal modes of the M^*m*+^(CH_3_CN)_*n*_ clusters (M^*m*+^ = Li^+^, Mg^2+^, Ca^2+^, or Zn^2+^ and *n* = 1–6) from that of an isolated CH_3_CN molecule plotted against *n*. (e–h) Similar plots of the isotropic Raman intensity-weighted average frequencies. (i–l) Corresponding values of the NCE calculated as ν_aniso_ – ν_iso_. Green: Li^+^, red: Mg^2+^, orange Ca^2+^, blue: Zn^2+^. Calculated at the (a,e,i) B3LYP/6-31+G­(2df,p), (b,f,j) HF/6-31+G­(2df,p), (c,g,k) MP2/6-31+G­(2df,p), and (d,h,l) MP3/6-31+G­(2df,p) levels. The 2CpM approximation is used in the calculations at MP3/6-31+G­(2df,p), and the values are extrapolated by a second polynomial fitting (color dotted lines). The black dotted horizontal line in panel a–h indicates the difference between the frequencies observed in the gas and liquid phases.^60^

Similar results are obtained at the HF level as shown in panel b and also for δν_iso_ as shown in panels e and f. However, at the MP2 level, significantly larger values of δν_IR_ and δν_iso_ with different forms of dependence on *n* are obtained as shown in panels c and g. To see which is more reliable, we conducted calculations at the MP3 level in the range of *n* feasible with the available computational resources. As shown in panels d and h, the MP3 calculations seem to support the values of δν_IR_ and δν_iso_ and their dependence on *n* obtained at the B3LYP level. The same conclusion is reached by referring to the calculated NCE values shown in panels i–l (discussed later). The reason for the peculiar behavior of the MP2 calculations is not clear at present.

The M^*m*+^···N radial distribution functions (RDFs) of the solutions of LiCl, MgCl_2_, CaCl_2_, and ZnCl_2_ dissolved in CH_3_CN solvent calculated by semiempirical QM/MM and classical MD simulations are shown in Figure 3a–d. It is seen that a strong single peak appears in each RDF, indicating that the CH_3_CN molecules are clustering around the ion with close M^*m*+^···N contacts also in solution. The peak is located at 2.06–2.21, 2.10–2.20, and 1.94–2.14 Å for Li^+^, Mg^2+^, and Zn^2+^ shown in panels a, b, and d, respectively. In the case of Ca^2+^ shown in panel c, the M^*m*+^···N distance is estimated differently by semiempirical QM/MM and classical MD simulations; the peak location is calculated as 2.25 and 2.20 Å in semiempirical QM/MM employing the DFTB3 and PM6 models, respectively, while it is calculated as 2.78 Å in classical MD. Comparing them with the M^*m*+^···N distances in the M^*m*+^(CH_3_CN)_*n*_ clusters calculated at the B3LYP, HF, MP2, and MP3 levels shown in Figure S2 in the Supporting Information, it is recognized that the M^*m*+^···N distance is underestimated and overestimated by semiempirical QM/MM and classical MD, respectively, in the case of Ca^2+^. For the other three ions, although the available semiempirical QM methods vary with the metal ions, both semiempirical QM/MM and classical MD provide rather stable estimates of the M^*m*+^···N distance. This point will be discussed later in Section 3.3 in relation to the estimated values of the NCE.

**Figure 3:**
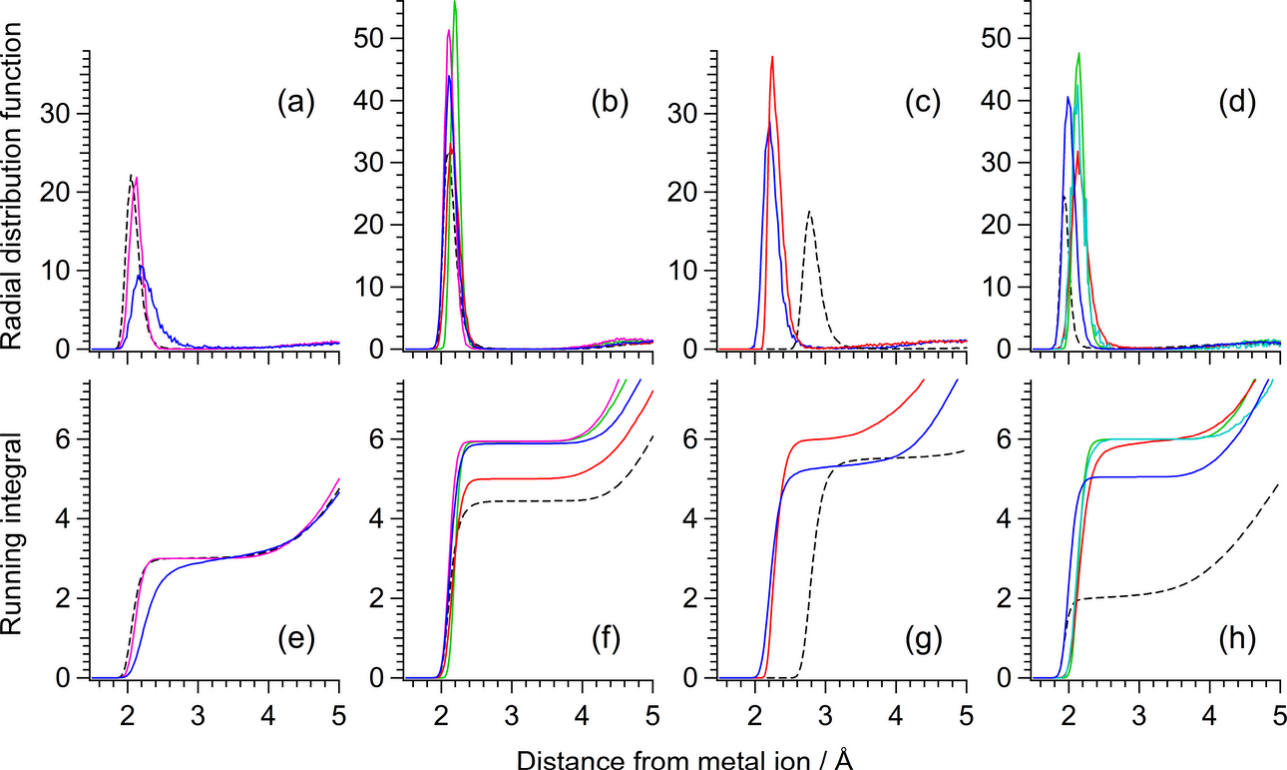
M^*m*+^···N radial distribution functions of the solutions of (a) LiCl, (b) MgCl_2_, (c) CaCl_2_, and (d) ZnCl_2_ dissolved in CH_3_CN solvent calculated by semiempirical QM/MM and classical MD simulations. Blue: PM6, light blue: PM3, green: AM1, pink: MNDO, red: DFTB3, black (broken line): classical MD. (e–h) Corresponding 4π*r* ^2^ρ-weighted running integrals.

The average number of CH_3_CN molecules in the first solvation shell around each M^*m*+^ ion (i.e., the coordination number) may be estimated from the 4π*r* ^2^ρ-weighted running integral of the RDF, which is shown in Figure 3e–h. It is seen that all the semiempirical QM/MM calculations provide rather converged estimates of the coordination number as the ordinate of the first plateau; it is ∼3 for Li^+^, and 5–6 for the three divalent cations. It is recognized that in the case of Zn^2+^, the coordination number is significantly underestimated as ∼2 by classical MD, possibly because a contact ion pair is formed at a shorter distance in this particular case than in the cases of the other three metal ions (the results not shown). For the solutions of the NTf_2_^–^ salts, the coordination number around Zn^2+^ is estimated as 5–6 by both semiempirical QM/MM and classical MD as shown in Figure S3 in the Supporting Information. However, in this case, the coordination number around Mg^2+^ seems somewhat underestimated by classical MD. The coordination numbers of *n* ∼3 for Li^+^ and ∼6 for Mg^2+^ and Zn^2+^ are in good agreement with those obtained experimentally by neutron diffraction, X-ray absorption near edge structure (XANES), and extended X-ray absorption fine structure (EXAFS) spectroscopies.^10–12^ It might be that the potential parameters used in classical MD need to be further improved. In the present study, the solvation structures obtained by the PM6 calculations, which are available for all four metal cations, will mainly be employed in the subsequent analysis.

Then, adopting the above-mentioned estimates of the coordination number as *n* and taking the corresponding values of δν_IR_ and δν_iso_ calculated at B3LYP shown in Figure 2a and e, the difference from the reference value of the gas-to-liquid frequency shift (−17.5 cm^–1^)^60^ is obtained as 40 and 42 cm^–1^ (δν_IR_ and δν_iso_, respectively) for Li^+^, 40 and 44 cm^–1^ for Mg^2+^, 26 and 30 cm^–1^ for Ca^2+^, and 43 and 48 cm^–1^ for Zn^2+^. [Applying the scale factor (0.9613) obtained from the calculated and observed frequencies of an isolated molecule, the above value should be interpreted as smaller by ∼1 cm^–1^; i.e., 39 and 41 cm^–1^ for Li^+^, 39 and 43 cm^–1^ for Mg^2+^, 25 and 29 cm^–1^ for Ca^2+^, and 42 and 47 cm^–1^ for Zn^2+^.] We consider these to be the calculated estimates that should be compared with the frequency shifts observed upon solvation of metal ions in liquid acetonitrile.

The polarized Raman spectra observed for the solutions of LiNTf_2_, Mg­(NTf_2_)_2_, Ca­(NTf_2_)_2_, and Zn­(NTf_2_)_2_ dissolved in the CD_3_CN solvent are shown in Figure 4. (As noted in Section 2.3, the solvent is deuterated to avoid spectral splitting due to Fermi resonance.) The strong band appearing at ∼2110 cm^–1^ arises from the CD_3_ symmetric stretching mode. The C≡N stretching band that originally exists in neat liquid (which is regarded as “free” from the metal ion) is located at 2266 cm^–1^, and upon solvation of salt, a band appears in the 2290–2310 cm^–1^ region, which is regarded as arising from the molecules “bound” to the metal ion. (See the concentration dependence observed for the solution of LiNTf_2_ shown in Figure S4 in the Supporting Information.) The frequency difference between the band of neat liquid and the newly appearing band is 25.0, 44.2, 26.0, and 44.4 cm^–1^ for solutions of LiNTf_2_, Mg­(NTf_2_)_2_, Ca­(NTf_2_)_2_, and Zn­(NTf_2_)_2_, respectively. Similar frequency differences were observed also (in many cases for the IR spectra) in previous studies.^8,9,27–39^ Comparing them with the above-mentioned computationally estimated values of these frequency differences, it is recognized that, except for the case of the Li^+^ salt, the observed values are reasonably well reproduced by the calculations. Considering the rather strong dependence of the frequency on *n* shown in Figure 2e,h (or Figure 2a,d for IR), we suggest that the observed large high-frequency shift of the newly appearing band means nearly full solvation of each metal ion by the C≡N groups of acetonitrile molecules. The reason for the overestimation in the case of the Li^+^ salt is not clear at present. Further sophisticated computations might be required for this system.

**Figure 4:**
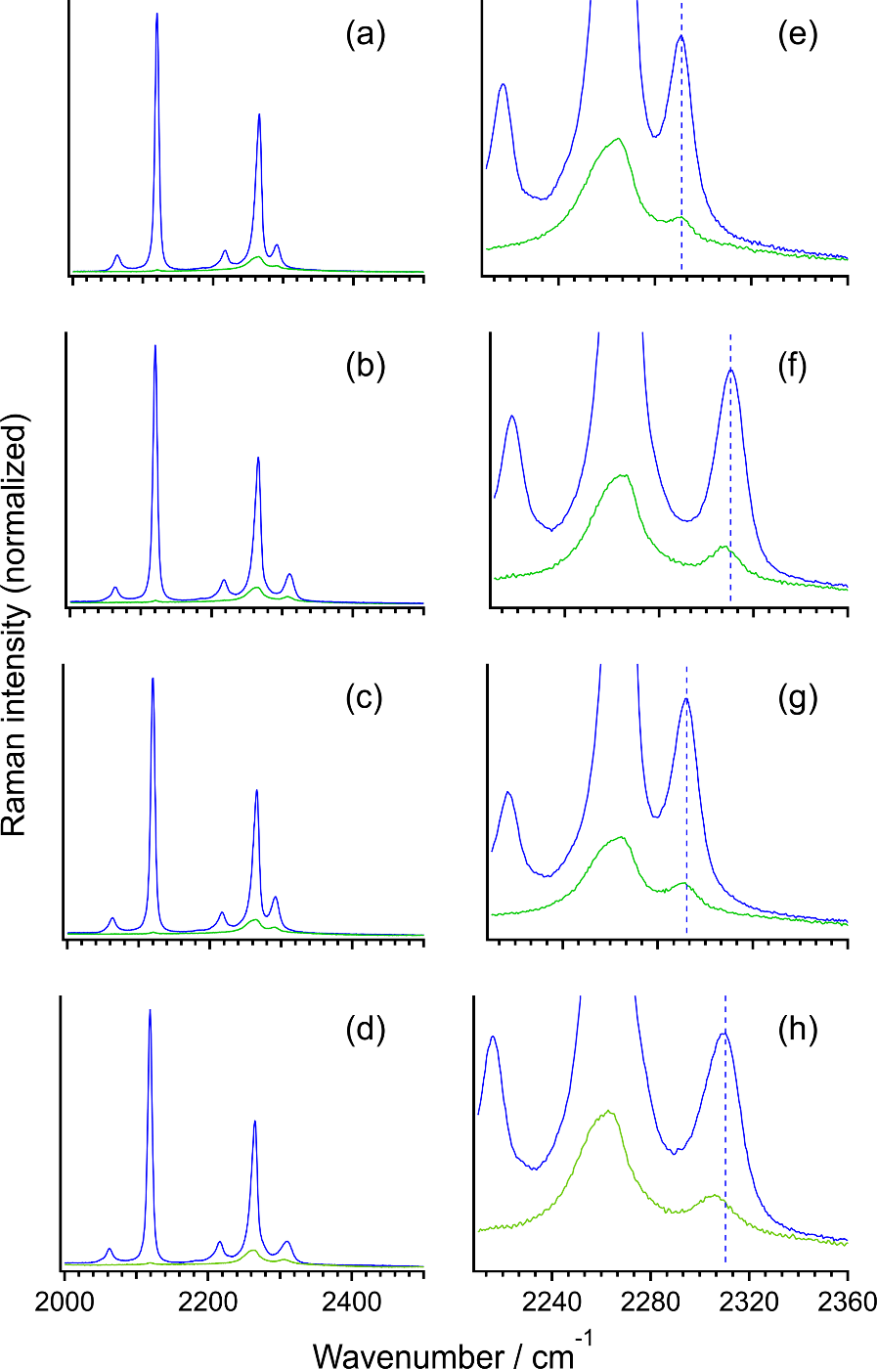
(a–d) VV-polarized (blue) and VH-polarized (green) Raman spectra observed in the 2000–2500 cm^–1^ region for the solutions of (a) LiNTf_2_, (b) Mg­(NTf_2_)_2_, (c) Ca­(NTf_2_)_2_, and (d) Zn­(NTf_2_)_2_ dissolved in CD_3_CN solvent (1.00 mol kg^–1^) at 298 K. (e–h) Expanded views in the 2210–2360 cm^–1^ region. The blue dotted vertical line in each panel indicates the peak position of the VV-polarized Raman band drawn as a guide to the eye.

High-frequency shifts of the C≡N stretching band are observed also for hydrogen-bonding systems^16–23^ and are explained by an electrostatic interaction model.^24–26,61^ Because the model includes both scalar and vector components,^24–26^ it is possible to reproduce both the high-frequency shifts induced by spatially nonuniform electrostatic perturbations arising from hydrogen-bond formation and the low-frequency shifts^15–20^ induced by sufficiently uniform electric fields in the N → C direction arising from dipolar solvation. It is also naturally derived from this model that the electric field in the opposite direction gives rise to a high-frequency shift^62,63^ if the field is sufficiently uniform. It would therefore be interesting to see to what extent this model applies to the cases of the M^*m*+^(CH_3_CN)_*n*_ clusters that are discussed in the present study. For this purpose, we have tried to estimate an effective electric charge of the metal ion in each cluster from the interaction-induced electron density changes. As an example, the electron density change induced by the formation of the Mg^2+^(CH_3_CN)_6_ cluster calculated at the B3LYP/6-31+G­(2df,p) level is shown in Figure 5a. Comparing it with the electron density change of one of the acetonitrile molecules (located on the left-hand side) by an electric charge of 0.2 *e* located at the position of the Mg^2+^ ion shown in Figure 5b, it is clearly recognized that these two electron density changes are quite similar to each other. More quantitatively, the similarity defined^64^ by the inner product of the two electron density changes within the rectangular box of *z* ≤ 1.4 Å and −2.0 Å ≤ *x*, *y* ≤ 2.0 Å shown with black dotted lines (where the origin is placed at the location of the N atom and the *z* axis is taken along the C≡N bond) is calculated as 0.853, which is considered to be reasonably high. [Higher values of similarity of 0.871, 0.902, and 0.880 are obtained for Li^+^(CH_3_CN)_4_, Ca^2+^(CH_3_CN)_6_, and Zn^2+^(CH_3_CN)_6_, respectively.] Then, based on the relative magnitudes of the two electron density changes, the effective electric charge is estimated as 0.967 *e*. The difference from the formal electric charge (2 *e*) is considered to arise from the direct effects of the other five acetonitrile molecules in the cluster (i.e., the effects of partially negatively charged N atoms clustering around the Mg^2+^ ion) and the reduction of the net electric charge of the Mg^2+^ ion due to charge transfer. Discussion on a way to decompose these two effects is deferred to later studies. Adopting this effective electric charge placed at the location of the Mg^2+^ ion and employing the coefficients of the electrostatic interaction model derived in ref 25, the frequency shift (from the frequency of an isolated molecule) is estimated as 31.7 cm^–1^. Dealing with the other clusters in a similar way, the effective electric charge and the frequency shift are estimated as 0.519 *e* and 20.0 cm^–1^ for Li^+^(CH_3_CN)_4_, 1.032 *e* and 20.6 cm^–1^ for Ca^2+^(CH_3_CN)_6_, and 1.014 *e* and 33.6 cm^–1^ for Zn^2+^(CH_3_CN)_6_. It is therefore concluded that the high-frequency shift observed for the C≡N stretching band that newly appears upon solvation of salt is largely explained (including higher frequency shifts for Mg^2+^ and Zn^2+^ compared with Li^+^ and Ca^2+^) by the electrostatic mechanism. The smaller high-frequency shift obtained for Ca^2+^ compared with the other two divalent ions arises from the longer M^*m*+^···N distance (2.504 Å for Ca^2+^, compared with 2.178, 2.169, and 2.244 Å for Mg^2+^, Zn^2+^, and Li^+^ as shown in Figure S2 in the Supporting Information) that makes the electrostatic perturbation on the CH_3_CN molecule weaker. This discussion does not exclude the possible existence of any other smaller effects that may be operating in inducing a high-frequency shift. A sign of the existence of those effects is recognized in the relation between the C≡N stretching force constants and the C≡N bond lengths shown in Figure S5 in the Supporting Information, where the points of the nearly fully solvated M^*m*+^(CH_3_CN)_*n*_ clusters are in the same tendency as those for hydrogen-bonded clusters and a molecule interacting with a positive partial charge, but those of the smaller M^*m*+^(CH_3_CN)_*n*_ clusters of divalent cations tend to deviate from it. Details of these smaller effects may deserve further studies.

**Figure 5:**
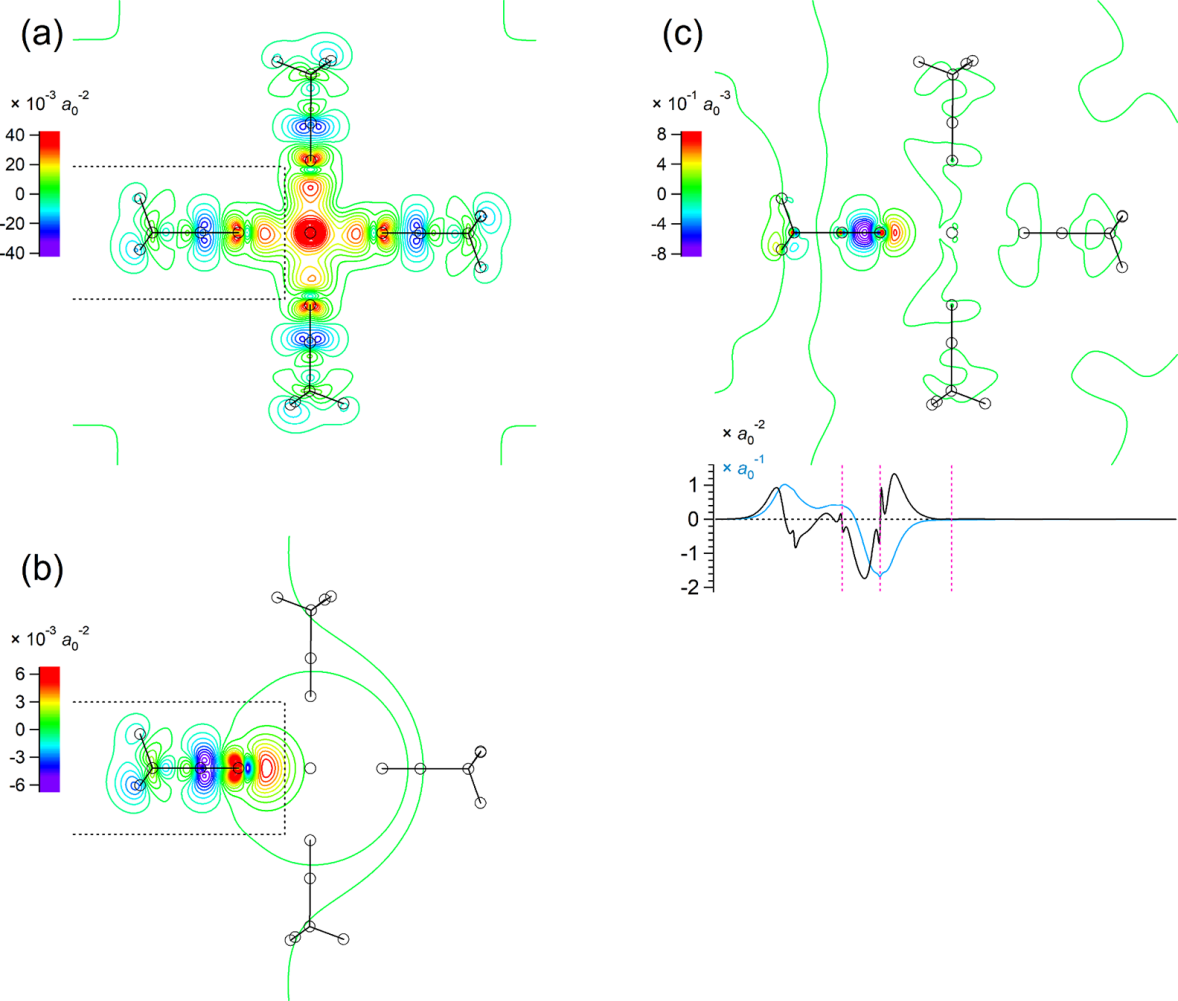
(a) Two-dimensional contour plot (integrated projection on the *yz* plane) of the electron density change δρ^(el)^(***r***) occurring upon the formation of the Mg^2+^(CH_3_CN)_6_ cluster calculated at the B3LYP/6-31+G­(2df,p) level. The *z* (horizontal) axis is taken along the C≡N bond of the CH_3_CN molecule located on the left-hand side. The contours are drawn with the interval of 0.32 × 10^–2^ *a*_0_^–2^ in the range from −8 to 8 × 10^–2^ *a*_0_^–2^, with the color code shown on the left-hand side. The locations of the atoms are indicated by black open circles. (Two molecules extended in the +*x* and −*x* directions are omitted for clarity.) The spatial region where the electron density changes are compared between panels a and b is indicated by black dotted lines. (b) The same type of plot of the electron density change δρ^(el)^(***r***) induced on the CH_3_CN molecule located on the left-hand side by an electric charge of 0.2 *e* placed at the location of the Mg^2+^ ion. The contours are drawn with the interval of 0.16 × 10^–2^ *a*_0_^–2^ in the range from −4 to 4 × 10^–2^ *a*_0_^–2^. (c) Two-dimensional contour plot (integrated projection on the *yz* plane), one-dimensional plot (integrated projection on the *z* axis, black), and its running integral (light blue) of the electron density derivative ∂ρ^(el)^(***r***)/∂*R*_CN_ calculated for a displacement along the C≡N stretching internal coordinate (*R*_CN_) of the CH_3_CN molecule located on the left-hand side in the Mg^2+^(CH_3_CN)_6_ cluster. The contours in the two-dimensional plot are drawn with an interval of 0.12 *a*_0_^–3^ in the range from −3 to 3 *a*_0_^–3^. In the one-dimensional plot, the positions of the C and N atoms of the C≡N bond of the vibrating molecule and the Mg^2+^ ion are indicated by pink dotted vertical lines.

### 3.3 Intermolecular Vibrational Coupling and Noncoincidence Effect

In each observed spectrum shown in Figure 4, the peak frequency of the VH-polarized (i.e., anisotropic) Raman component of the C≡N stretching band is slightly lower than that of the VV-polarized Raman component (which is essentially equal to the isotropic Raman component because the anisotropic component is very weak in the present case). The magnitude of this negative NCE is small [δν_NCE_ (≡ ν_aniso_ – ν_iso_) = −0.5, −3.0, −1.7, and −4.1 cm^–1^ for the newly appearing band of the solutions of LiNTf_2_, Mg­(NTf_2_)_2_, Ca­(NTf_2_)_2_, and Zn­(NTf_2_)_2_, respectively] but seems to be qualitatively consistent with the same phenomenon observed for the solutions of some electrolytes dissolved in acetone and carbonate esters.^46–48^ From the DFT and ab initio MO calculations on the cluster species shown in Figure 2i–l, this negative NCE is also predicted except at the MP2 level. (This result supports the conclusion made in Section 3.2 that the calculations at the B3LYP level are more reliable.) The order of the magnitude of this NCE (in absolute value) is predicted as Li^+^ < Ca^2+^ < Mg^2+^ < Zn^2+^, which is fully consistent with the observed result. We consider that this result is consistent with the nearly full solvation structure of each metal ion by the C≡N groups of acetonitrile molecules that is suggested in the discussion in Section 3.2.

The NCE arises from intermolecular vibrational coupling,^41–45^ and it is discussed that this coupling is important also for molecules clustering around a metal ion.^46–48,65,66^ Considering that the 2CpM rather than 1CpM approximation works well in calculating the C≡N stretching frequencies as shown in Section 3.1, which means that the *intra*molecular coupling between the vibrations along the C≡N and C–C stretching internal coordinates plays an essential role in them, it would be interesting to see to what extent the *inter*molecular coupling can be described only by the vibrations along the C≡N stretching internal coordinates. For this purpose, the NCE values calculated by neglecting the vibrations along the C–C stretching internal coordinates in the *inter*molecular coupling are compared with those derived from the full treatment within the 2CpM approximation in Figure 6a–c (calculated at B3LYP, HF, and MP3, respectively). It is seen that, in the range of |δν_NCE_| < 6 cm^–1^, the *inter*molecular coupling related to the C–C stretching internal coordinates is indeed safely neglected. The deviation becomes larger beyond this range, but it seems that it is not much scattered.

**Figure 6:**
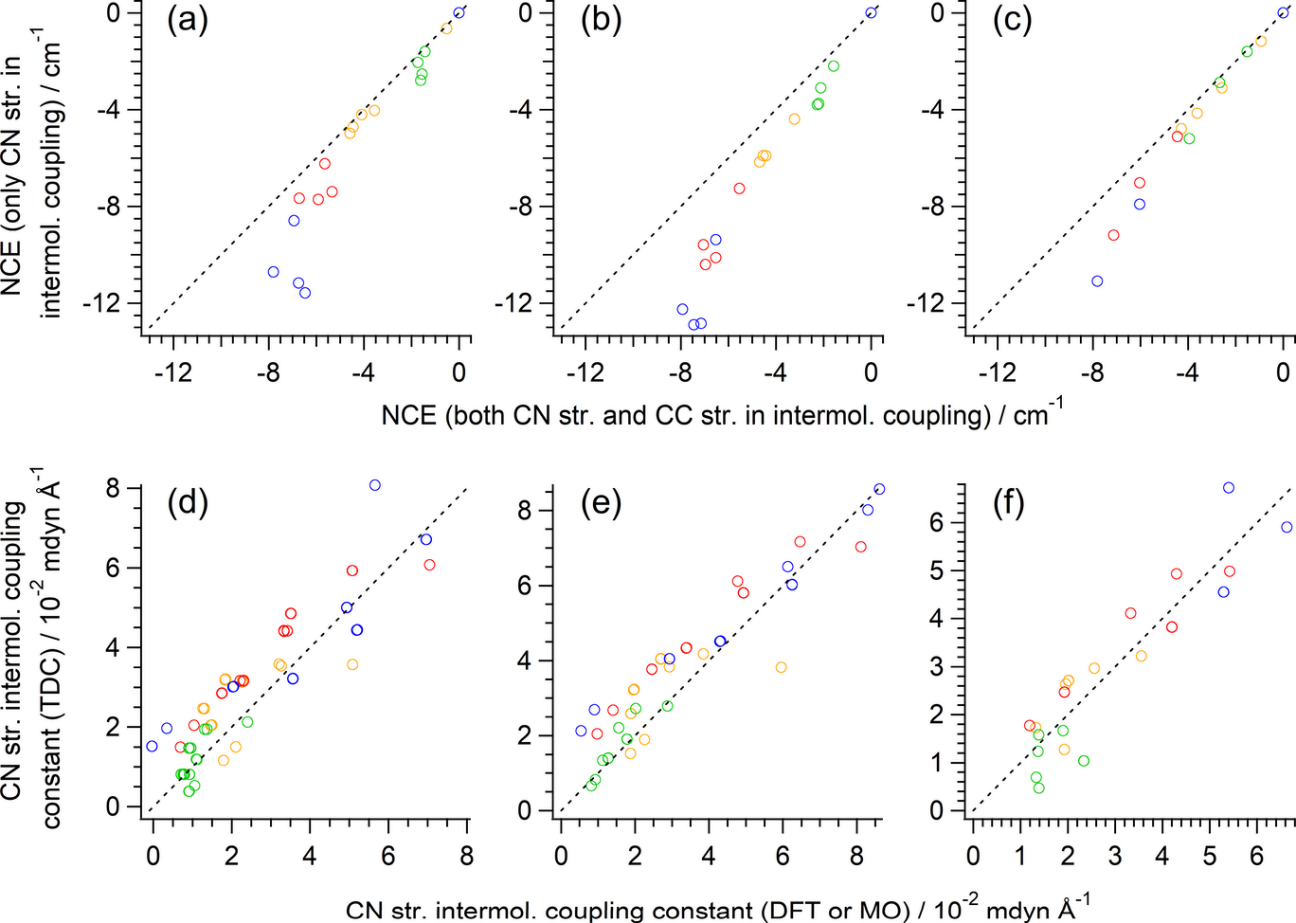
(a–c) NCE values of the C≡N stretching modes of the M^*m*+^(CH_3_CN)_*n*_ clusters (M^*m*+^ = Li^+^, Mg^2+^, Ca^2+^, or Zn^2+^ and *n* = 1–6) calculated by including only the C≡N stretching internal coordinates for the intermolecular vibrational couplings plotted against the values calculated with the full treatment within the 2CpM approximation. (d–f) Intermolecular vibrational coupling constants of the C≡N stretching internal coordinates based on the transition dipole coupling (TDC) mechanism obtained by placing the transition dipole at the C atom of the C≡N bond in each molecule plotted against those directly obtained by the DFT or MO method. Green: Li^+^, red: Mg^2+^, orange Ca^2+^, blue: Zn^2+^. Calculated at the (a,d) B3LYP/6-31+G­(2df,p), (b,e) HF/6-31+G­(2df,p), and (c,f) MP3/6-31+G­(2df,p) levels. The black dotted line in each panel indicates the line of gradient unity passing through the origin drawn as a guide to the eye.

It is known^42–48,67^ that, in many cases, the TDC mechanism plays an important role in intermolecular vibrational coupling. To apply this model, one important point is to decide where the transition dipole (proportional to the dipole derivative in the vibrational coupling) should be located in each molecule. To see the spatial characteristics of the dipole derivative ∂μ/∂*R*_CN_ of the C≡N stretch (*R*_CN_), electron density derivative ∂ρ^(el)^(***r***)/∂*R*_CN_ is calculated for the Mg^2+^(CH_3_CN)_6_ cluster (as an example case) and is shown in Figure 5c. From the two-dimensional plot shown on the upper part, it might seem that the main amplitudes are localized around the N atom, but in fact, as recognized from the one-dimensional plot (black line) and its running integral (light blue line) shown on the lower part, the main amplitudes are extended over the whole molecule. Consistent with this, if we locate the dipole derivative ∂μ/∂*R*_CN_ at the C atom of the C≡N bond in each molecule in calculating the intermolecular vibrational coupling constants according to the TDC mechanism, then it is possible to obtain reasonably good agreement with those derived directly from DFT or ab initio MO calculations, as shown in Figure 6d–f. In contrast, if we locate it at the C≡N bond center in each molecule, the TDC mechanism significantly overestimates the coupling, as shown in Figure S6 in the Supporting Information, because the dipole derivatives of difference molecules in a cluster are located too close to each other.

If one tries to estimate the values of NCE by including the effects of thermal disorder in the solvation structures, it is required to evaluate the intermolecular vibrational coupling with an appropriate model and combine it with solution structures obtained by a certain kind of MD simulation.^43,68–70^ In the present study, we adopt the scheme of evaluating the intermolecular vibrational coupling by the TDC of the C≡N stretching internal coordinates, with the dipole derivative ∂μ/∂*R*_CN_ of each molecule taken as parallel to the C≡N bond and located on the C atom of this bond, with its magnitude taken from the calculations at the B3LYP/6-31+G­(2df,p) level and scaled to reproduce the DFT-derived coupling constants (Figure 6d), and combining it with the solution structures obtained by MD simulations. The Raman tensor of the C≡N stretch of each molecule is assumed to be axially symmetric with respect to the C≡N bond, with the ratio of the magnitudes of its principal axes being set as 3:1:1 to approximately reproduce the observed depolarization ratio. The scaled magnitudes of the dipole derivative, as well as the average intramolecular force constants (scaled by the square of 0.9613) to be used for constructing the force constant matrix within the 2CpM approximation, are shown in Table S1 in the Supporting Information. (Here, the values for *n* = 7 are also included for Ca^2+^.) The spectra simulated by employing the PM6 method of semiempirical QM/MM MD (which is adopted because it is available for all the four metal ions) are shown in the upper eight panels of Figure 7. The frequency positions (first moments) and the values of the NCE (defined as ν_aniso_ – ν_iso_) are summarized in Table S2 in the Supporting Information. (For comparison, the observed frequencies are summarized in Table S3.) It is seen that a negative NCE (ν_aniso_ < ν_iso_) is calculated in all cases, with its magnitude being smallest for Li^+^ and largest for Zn^2+^, in agreement with the experiment referred to at the beginning of this section. More quantitatively, however, the magnitude of the NCE is, overall, somewhat overestimated. The deviation is particularly large for Ca^2+^, where the magnitude of the NCE is predicted to be larger than that for Mg^2+^. This is probably because the Ca^2+^···N distance is significantly underestimated by semiempirical QM/MM MD as discussed in Section 3.2, leading to mutually too close locations of the dipole derivatives of different molecules in a cluster and, hence, too strong intermolecular vibrational coupling. Indeed, if we adopt the liquid structures simulated by classical MD, where the Ca^2+^···N distance is in turn slightly overestimated, the predicted magnitude of the NCE is reduced by about 40% and becomes in better agreement with experiment as shown in Figure 7l. One problem with regard to the simulations based on classical MD liquid structures is that, for Mg^2+^, the band is split into two components as shown in Figure 7g,h, and the predicted magnitude of NCE is too large. This is probably because the coordination number is significantly underestimated, as shown in Figure S3f in the Supporting Information.

**Figure 7:**
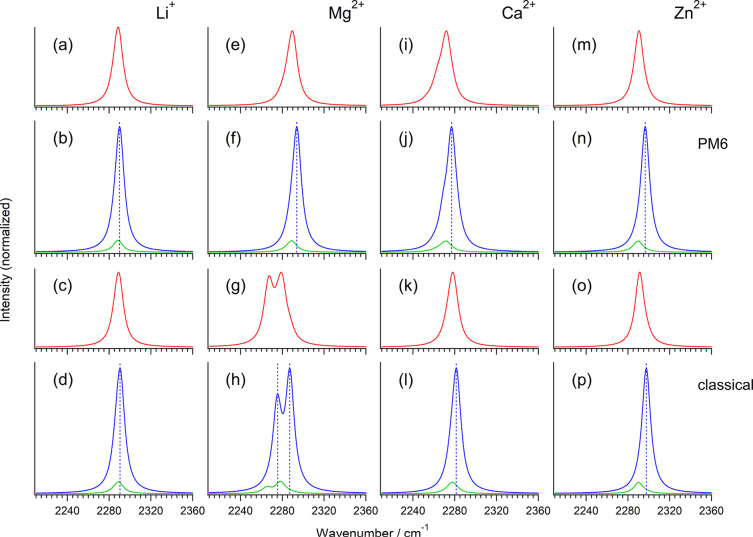
IR, isotropic Raman, and anisotropic Raman C≡N stretching bands (red, blue, and green, respectively) arising from the solvation structure around each metal ion of the solutions of (a–d) LiNTf_2_, (e–h) Mg­(NTf_2_)_2_, (i–l) Ca­(NTf_2_)_2_, and (m–p) Zn­(NTf_2_)_2_ dissolved in CH_3_CN solvent simulated on the basis of the PM6 semiempirical QM/MM MD (upper eight panels) and classical MD (lower eight panels) liquid structures combined with the 2CpM approximation and the TDC mechanism. A Lorentzian band with a half-width at half-maximum of 5 cm^–1^ is convoluted in each spectrum. The blue dotted vertical line in each Raman spectrum indicates the peak position of the isotropic Raman band drawn as a guide to the eye.

The comparison of the observed and calculated values of the NCE indicates, therefore, that the slightly negative NCE observed for the band that appears newly in the C≡N stretch region upon solvation of electrolytes, with the magnitude in the order of Li^+^ < Ca^2+^ < Mg^2+^ < Zn^2+^, is consistent with the nearly full solvation structure of each metal ion by the C≡N groups of acetonitrile molecules, with the coordination number (*n*) and the typical M^*m*+^···N distance (*d*) of *n* = 3–4 and *d* = 2.1–2.2 Å for Li^+^, *n* = 5–6 and *d* = 2.1–2.2 Å for Mg^2+^, *n* = 5–6 and *d* ∼ 2.5 Å for Ca^2+^, and *n* = 5–6 and *d* = 1.9–2.1 Å for Zn^2+^. It may also be said that, for performing spectral simulations based on MD liquid structures, it would be important to select an appropriate method of QM in QM/MM MD or a sophisticated potential model in classical MD.

## 4 Concluding Remarks

The results obtained in the present study have provided (as we consider) a basically consistent view of the relation among the solvation structures, the spectral features in the C≡N stretching region, and the intermolecular interactions of acetonitrile electrolyte solutions. The C≡N stretching vibrations of the acetonitrile molecules clustering around each metal ion M^*m*+^ (= Li^+^, Mg^2+^, Ca^2+^, or Zn^2+^) are mutually coupled, and this coupling gives rise to a negative NCE (Figures 2i–l, 4, and 7). It is observed that its magnitude increases in the order of Li^+^ < Ca^2+^ < Mg^2+^ < Zn^2+^ (in absolute value), and this order is reproduced by quantum chemical calculations. Similar to the cases of the electrolyte solutions of acetone and carbonate esters,^46–48^ the negative sign of this NCE is reasonably well explained by the TDC mechanism and the “clustering” nature of the structural configurations around a metal ion (which is totally different from those in neat liquid). As a result, it is possible to perform spectral simulations based on this TDC mechanism and the solvation structures extracted from MD simulations (Figure 7). From quantum chemical calculations, it has also been derived that the frequency shifts of the C≡N stretching mode induced by the M^*m*+^···N≡C interactions depend rather strongly on the coordination number (Figure 2a–h). Therefore, the observed high-frequency shift, together with the above-mentioned negative NCE, is consistent with a nearly full solvation around each metal ion, which is supported by MD simulations (Figure 3). It has also been shown that this high-frequency shift is largely of electrostatic origin and is explainable by a model^25^ that takes into account the spatially nonuniform nature of the electrostatic potentials and fields generated around each metal ion. With regard to a technical aspect of theoretical analysis, it has been shown that the 2CpM rather than 1CpM approximation can safely be used in calculating the C≡N stretching frequencies of acetonitrile in the M^*m*+^(CH_3_CN)_*n*_ clusters (Figure 1), especially at a high theoretical level that demands a large amount of computational resources.

Vibrational modes of solvent molecules, if they are sufficiently sensitive via frequency shifts and spectral phenomena arising from vibrational coupling, are useful probes of intermolecular interactions and structural configurations. The C≡N stretching mode is one of such useful probes. Because it is intrinsically present in a nitrile compound, it can be basically applied to systems of any concentration without extra perturbations. In that sense, the information derived in this study on the mechanisms that give rise to some spectral features would be helpful in those applications.

## Supplementary Material



## Notes

Some supplementary figures and tables showing the performance of the 1CpM approximation, some structural and vibrational properties in the M^*m*+^(CH_3_CN)_*n*_ clusters, radial distribution functions of the solutions of the NTf_2_^–^ salts, concentration dependence of the observed VV-polarized spectra, and a summary of the simulated and observed frequencies of the polarized Raman bands (PDF)
