# Supplementary material for: Frequency Shifts and Noncoincidence Effect of the CN Stretching Modes and the Solvation Structures of Acetonitrile Electrolyte Solutions
Source: J Phys Chem B. 2025 Jul 7;129(28):7270–81. doi: 10.1021/acs.jpcb.5c01894 (PMC12434666; doi:10.1021/acs.jpcb.5c01894)
Supplement: Supplementary file 1 [file jp5c01894_si_001.pdf]

Supporting Information:

Frequency Shifts and Noncoincidence Effect of the CN Stretching Modes and the Solvation Structures of Acetonitrile Electrolyte Solutions

Miyu Hirose,[1] Yukichi Kitamura,[1]* Hideaki Shirota,[2]* and Hajime Torii[1,3]*

[1] *Applied Chemistry and Biochemical Engineering Course, Department of Engineering, Graduate School of Integrated Science and Technology, Shizuoka University, 3-5-1 Johoku, Chuo-ku, Hamamatsu 432-8561, Japan*

[2] *Department of Chemistry, Chiba University, 1-33 Yayoi, Inage-ku, Chiba 263-8522, Japan*

[3] *Department of Optoelectronics and Nanostructure Science, Graduate School of Science and Technology, Shizuoka University, 3-5-1 Johoku, Chuo-ku, Hamamatsu 432-8561, Japan*

* Corresponding authors.

E-mail: kitamura.yuhkichi@shizuoka.ac.jp, shirota@faculty.chiba-u.jp, torii.hajime@shizuoka.ac.jp

Supplementary Figures

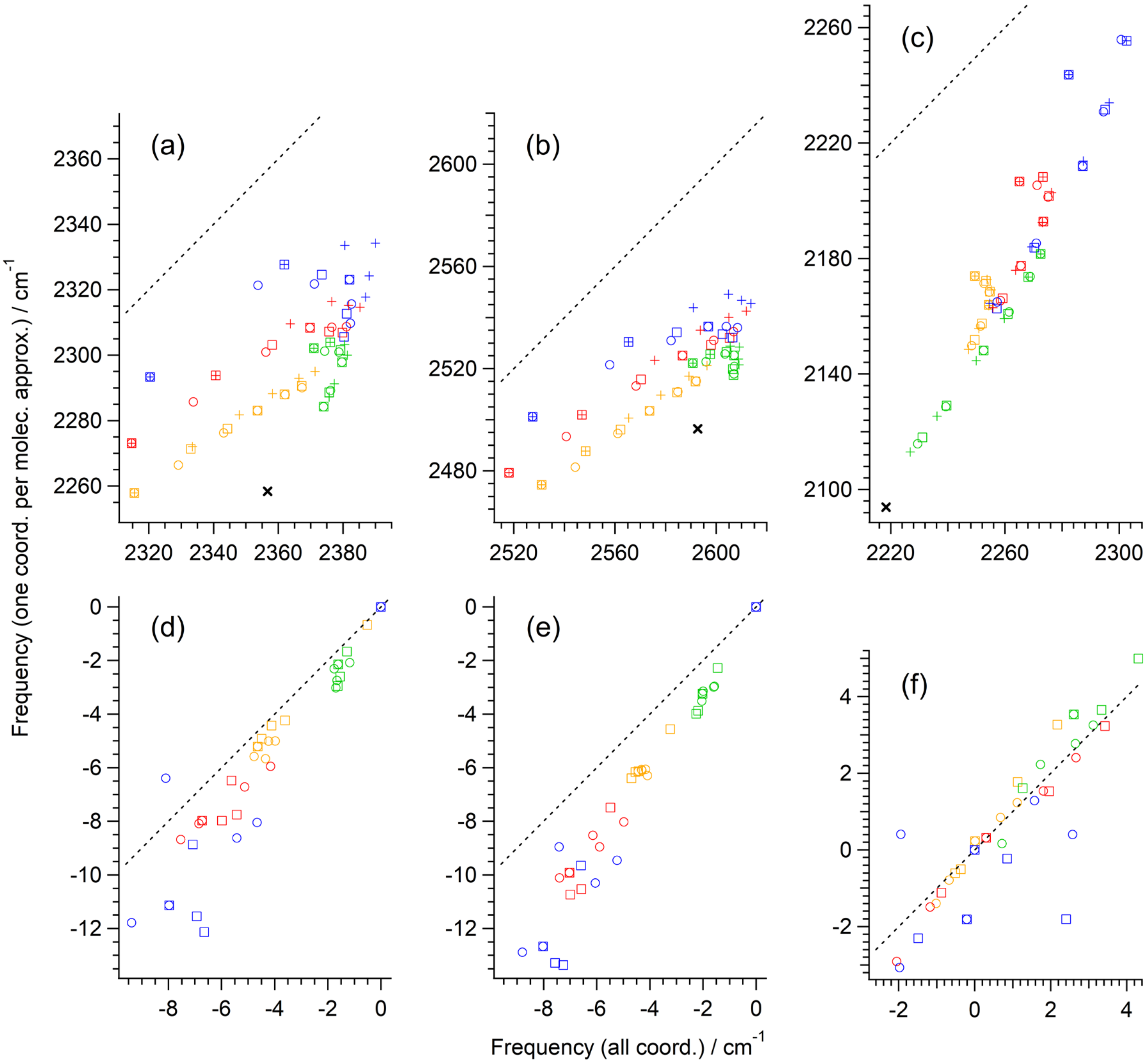

Figure S1. (a–c) Intensity-weighted average frequencies of the C≡N stretching normal modes of the $M^{m+}(CH_3CN)_n$ clusters (M^{m+} = Li^+, Mg^{2+}, Ca^{2+}, or Zn^{2+} and n = 1–6) and an isolated CH_3CN molecule calculated by including only the C≡N stretching internal coordinate(s) (1CpM approximation) plotted against those calculated by including all coordinates. (d–f) Corresponding values of the NCE calculated as $\nu_{IR} - \nu_{iso}$ or $\nu_{aniso} - \nu_{iso}$. Circle: IR (or $\nu_{IR} - \nu_{iso}$), square: anisotropic Raman (or $\nu_{aniso} - \nu_{iso}$), +: isotropic Raman, green: Li^+, red: Mg^{2+}, orange Ca^{2+}, blue: Zn^{2+}, × (black): isolated molecule. Calculated at the (a,d) B3LYP/6-31+G(2df,p), (b,e) HF/6-31+G(2df,p), and (c,f) MP2/6-31+G(2df,p) levels. The black dotted line in each panel indicates the line of gradient unity passing through the origin drawn as the guide to the eye.

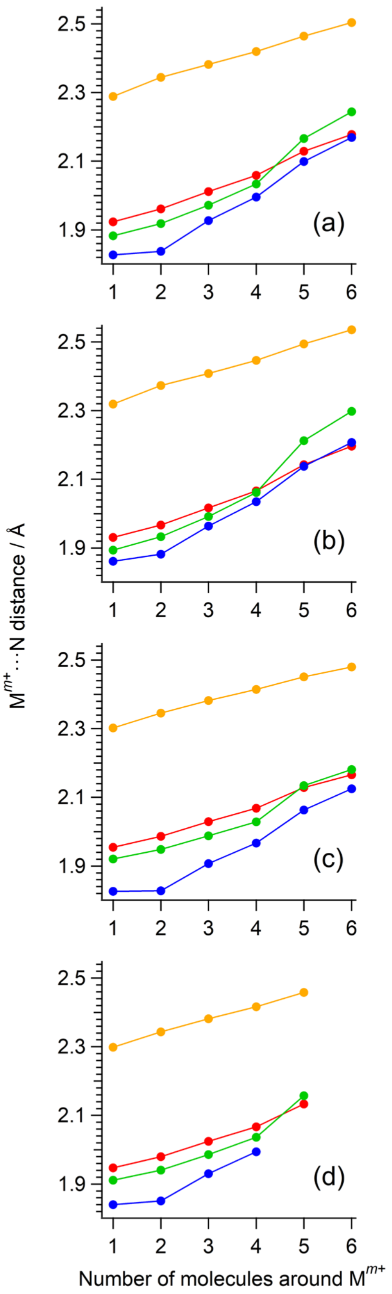

Figure S2. Average $M^{m+}\cdots N$ distance in the $M^{m+}(CH_3CN)_n$ clusters (M^{m+} = Li^+, Mg^{2+}, Ca^{2+}, or Zn^{2+} and n = 1–6; green: Li^+, red: Mg^{2+}, orange Ca^{2+}, blue: Zn^{2+}) calculated at the (a) B3LYP/6-31+G(2df,p), (b) HF/6-31+G(2df,p), (c) MP2/6-31+G(2df,p), and (d) MP3/6-31+G(2df,p) levels.

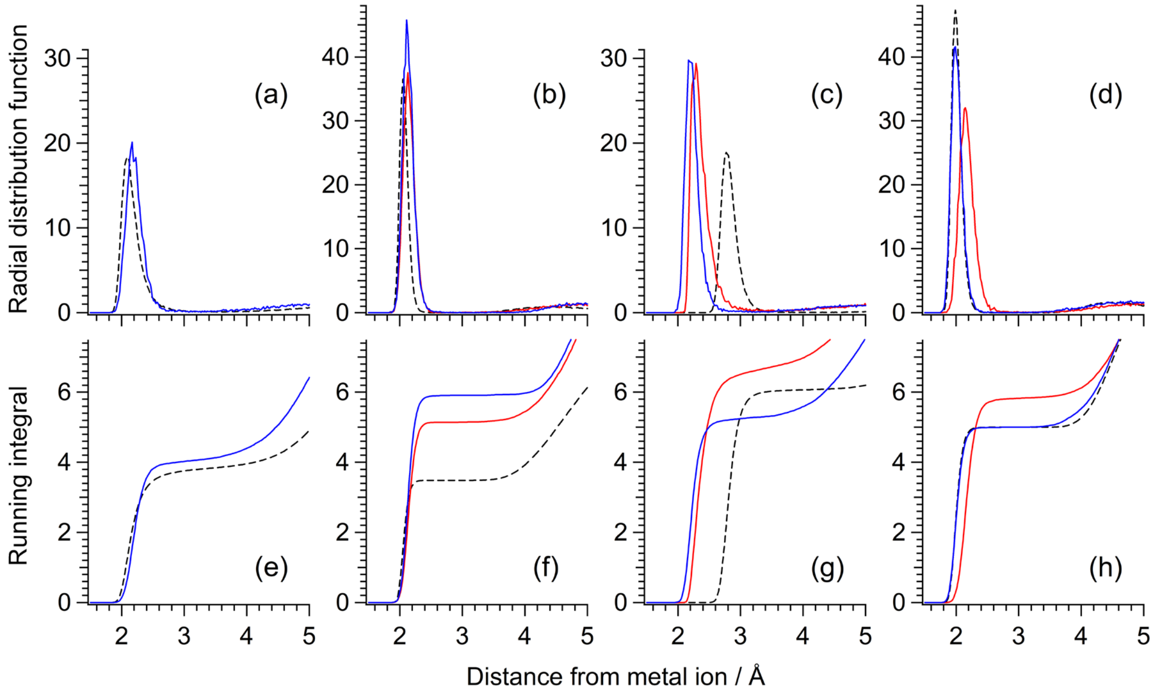

Figure S3. $M^{m+}\cdots N$ (of CH_3CN) radial distribution functions of the solutions of (a) $LiNTf_2$, (b) $Mg(NTf_2)_2$, (c) $Ca(NTf_2)_2$, and (d) $Zn(NTf_2)_2$ dissolved in CH_3CN solvent calculated by semi-empirical QM/MM and classical MD simulations. Blue: PM6, red: DFTB3, black (broken line): classical MD. (e–h) Corresponding $4\pi r^2\rho$-weighted running integrals.

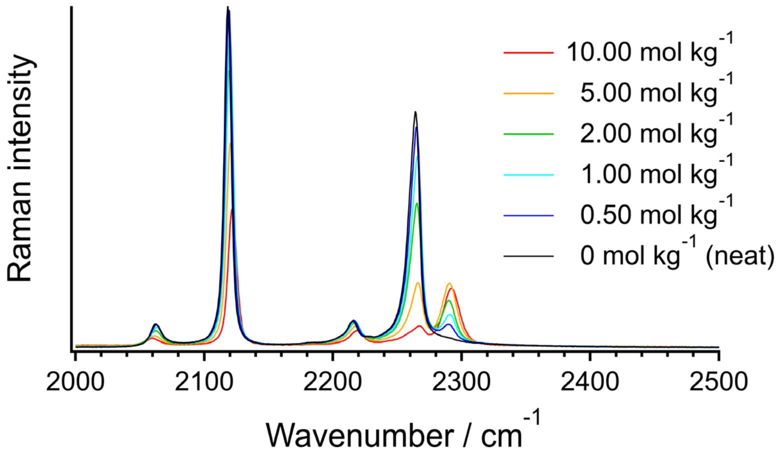

Figure S4. Concentration dependence of the VV-polarized Raman spectrum observed in the 2000–2500 cm^{-1} region for the solution of $LiNTf_2$ dissolved in CD_3CN solvent at 298 K.

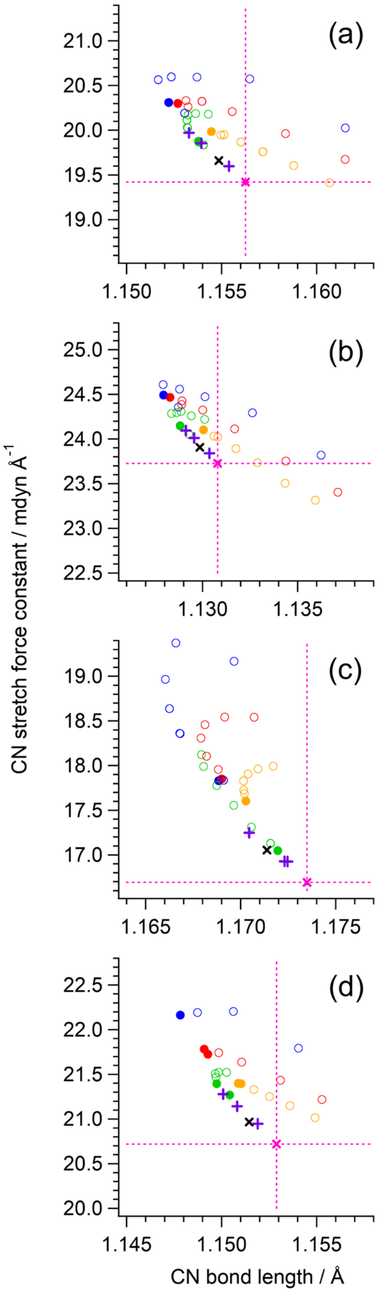

Figure S5. Plot of the C≡N stretching force constant on the internal-coordinate basis against the C≡N bond length calculated at the (a) B3LYP/6-31+G(2df,p), (b) HF/6-31+G(2df,p), (c) MP2/6-31+G(2df,p), and (d) MP3/6-31+G(2df,p) levels for the $M^{m+}(CH_3CN)_n$ clusters ($M^{m+} = Li^+$, Mg^{2+}, Ca^{2+}, or Zn^{2+} and n = 1–6; green: Li^+, red: Mg^{2+}, orange Ca^{2+}, blue: Zn^{2+}; filled circles for the largest n at the respective theoretical levels and open circles for others), the 1:1 hydrogen-bonded clusters of CH_3CN with urea, 2,2,2-trifluoroethanol, and 1,1,1,3,3,3-hexafluoro-2-propanol (purple +) taken from ref 25, and the CH_3CN molecule interacting with a positive partial charge of 0.2 e placed at 2.0 Å on the line extended from the C≡N bond (black ×). The point of an isolated CH_3CN molecule is indicated with pink × (pink vertical and horizontal lines are added for clarity).

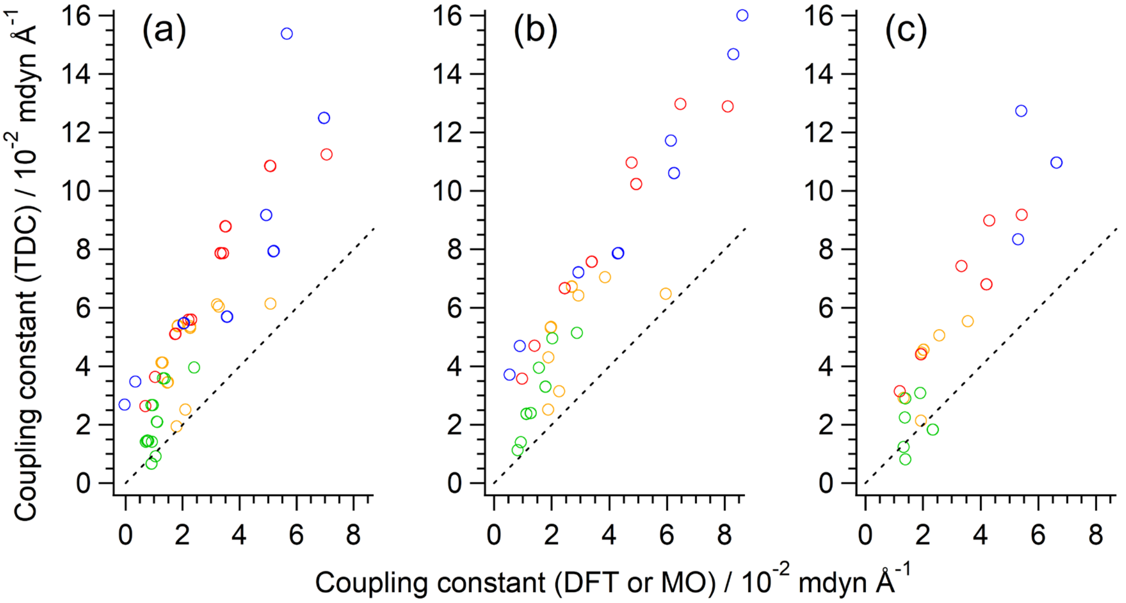

Figure S6. (a–c) Intermolecular vibrational coupling constants of the C≡N stretching internal coordinates based on the transition dipole coupling (TDC) mechanism obtained by placing the transition dipole at the C≡N bond center in each molecule plotted against those directly obtained by the DFT or MO method for the $M^{m+}(CH_3CN)_n$ clusters (M^{m+} = Li^+, Mg^{2+}, Ca^{2+}, or Zn^{2+} and n = 1–6). Green: Li^+, red: Mg^{2+}, orange Ca^{2+}, blue: Zn^{2+}. Calculated at the (a) B3LYP/6-31+G(2df,p), (b) HF/6-31+G(2df,p), and (c) MP3/6-31+G(2df,p) levels. The black dotted line in each panel indicates the line of gradient unity passing through the origin drawn as the guide to the eye.

Supplementary Tables

Table S1. Average magnitude of the dipole derivative $\partial\boldsymbol{\mu}/\partial R_{CN}$ of the C≡N stretch and the intramolecular force constants of the C≡N and C–C stretches in the $M^{m+}(CH_3CN)_n$ clusters [a]

		dipole derivative / D Å^{-1}		force constant [b] / mdyn Å^{-1}		
metal ion	number of molecules (n)	original	scaled	C≡N str. diag.	C–C str. diag.	C≡N str./C–C str. intramol. coupling
Li^+	2	4.959	5.282	18.654	4.900	0.401
	3	4.196	3.300	18.640	4.889	0.331
	4	3.523	2.592	18.587	4.879	0.276
	5	2.885	2.249	18.436	4.860	0.206
	6	2.448	2.036	18.366	4.855	0.171
Mg^{2+}	2	8.586	9.253	18.447	5.007	0.768
	3	7.479	6.581	18.674	4.946	0.647
	4	6.484	5.164	18.781	4.909	0.552
	5	5.433	4.021	18.762	4.869	0.448
	6	4.694	3.315	18.760	4.854	0.386
Ca^{2+}	2	7.643	8.094	18.116	4.880	0.544
	3	6.848	6.114	18.259	4.860	0.475
	4	6.128	4.995	18.360	4.850	0.421
	5	5.351	4.206	18.433	4.840	0.369

	6	4.768	3.729	18.468	4.838	0.328
	7	4.105	3.012	18.466	4.830	0.283
Zn^{2+}	2	9.312	7.793	19.012	5.109	0.978
	3	7.634	6.986	19.031	4.975	0.732
	4	6.384	5.410	19.034	4.912	0.585
	5	5.401	4.199	18.866	4.863	0.454
	6	4.713	3.500	18.770	4.842	0.376

[a] Calculated at the B3LYP/6-31+G(2df,p) level.

[b] Scaled by the square of 0.9613 (obtained from the calculated and observed frequencies of an isolated CH_3CN molecule).

Table S2. First moments of the IR, isotropic Raman, and anisotropic Raman C≡N stretching bands arising from the solvation structure around each metal ion of the solutions of $LiNTf_2$, $Mg(NTf_2)_2$, $Ca(NTf_2)_2$, and $Zn(NTf_2)_2$ dissolved in CH_3CN solvent and the value of the corresponding noncoincidence effect ($\equiv \nu_{sniso} - \nu_{iso}$) simulated on the basis of the PM6 semi-empirical QM/MM and classical MD liquid structures combined with the 2CpM approximation and the TDC mechanism.

		first moment / cm^{-1}			
MD	metal ion	IR	iso	aniso	NCE / cm^{-1}
PM6	Li^+	2288.4	2289.7	2288.3	−1.4
	Mg^{2+}	2288.8	2293.1	2288.1	−4.9
	Ca^{2+}	2270.2	2275.0	2269.6	−5.4
	Zn^{2+}	2290.4	2296.3	2289.7	−6.6
classical	Li^+	2288.7	2290.1	2288.7	−1.3
	Mg^{2+}	2274.7	2281.2	2273.9	−7.2
	Ca^{2+}	2277.8	2280.7	2277.3	−3.4
	Zn^{2+}	2291.6	2297.5	2290.6	−6.8

Table S3. Frequency positions of the VV- and VH-polarized Raman C≡N stretching bands that newly appear upon solvation of $LiNTf_2$, $Mg(NTf_2)_2$, $Ca(NTf_2)_2$, and $Zn(NTf_2)_2$ in CD_3CN solvent and the value of the corresponding noncoincidence effect.

solute	VV-polarized / cm^{-1}	VH-polarized / cm^{-1}	NCE / cm^{-1}
$LiNTf_2$	2290.5	2290.0	−0.5
$Mg(NTf_2)_2$	2310.3	2307.3	−3.0
$Ca(NTf_2)_2$	2291.8	2290.1	−1.7
$Zn(NTf_2)_2$	2309.4	2305.3	−4.1
